# Arsenic uptake and accumulation in bean and lettuce plants at different developmental stages

**DOI:** 10.1007/s11356-023-30593-7

**Published:** 2023-11-02

**Authors:** Sirat Sandil, Gyula Záray, Anett Endrédi, Anna Füzy, Tünde Takács, Mihály Óvári, Péter Dobosy

**Affiliations:** 1 Cooperative Research Centre of Environmental Sciences, Eötvös Loránd University, Pázmány Péter sétány 1/A, Budapest, H-1117 Hungary; 2 Institute of Aquatic Ecology, HUN-REN Centre for Ecological Research, Karolina út 29-31, Budapest, H-1113 Hungary; 3 Institute for Soil Sciences, HUN-REN Centre for Agricultural Research, Herman Ottó út 15, Budapest, H-1022 Hungary; 4 Nuclear Security Department, HUN-REN Centre for Energy Research, Konkoly-Thege Miklós út 29-33, Budapest, H-1121 Hungary

**Keywords:** Vegetables, Arsenic, Irrigation, Developmental stages, Transfer factor, Health risk assessment

## Abstract

The pattern of arsenic (As) uptake at different developmental stages in plants and its consequent influence on the growth of plants was investigated in bean and lettuce. Further, the human health risk from the consumption of these As-laced vegetables was determined. The irrigation water was contaminated with As at concentrations of 0.1, 0.25, and 0.5 mg/L. The As concentration in the plant parts (root, stem, leaves, and flower/fruit) was determined in bean at the young, flowering, and fruiting stages and lettuce at the young and mature stages. At the different growth stages, As had an impact on the biomass of bean and lettuce plant parts, but none of the biomass changes were significant (*p*>0.05). The increase in As concentration of the irrigation water elevated the As concentration of plant parts of both plants at all growth stages, with the exception of the bean fruit. The As concentration in the developmental stages was in the order: lettuce (young>mature) and bean (fruiting>young>flowering). In lettuce, the transfer factor was higher at the young stage (0.09–0.19, in the control and 0.1 mg/L As treatment), while in bean, it was highest at the flowering stage (0.09–0.41, in all treatments). In the edible part, lettuce possessed substantially elevated As concentrations (0.30, 0.61, and 1.21 mg/kg DW) compared to bean (0.008, 0.005, and 0.022 mg/kg DW) at As treatments of 0.1, 0.25, and 0.5 mg/L, respectively, and posed significant health risks at all applied As concentrations.

**Supplementary Information:**

The online version contains supplementary material available at 10.1007/s11356-023-30593-7.

## Introduction

Arsenic (As), a naturally occurring metalloid, has obtained the spotlight as a contaminant of concern due to its ubiquitous presence in various environments all over the world. Despite being a scarce element in the environment, As is a significant soil and water pollutant detrimental to plants, animals, and human beings. It has been labeled by the International Agency for Research on Cancer (IARC 2012) as a Group I carcinogen. Geogenic As contamination of the groundwater poses an environmental and health hazard in about 108 countries worldwide, endangering over 220–230 million people (Shaji et al. 2021; Muzaffar et al. 2023). Arsenic in the groundwater is primarily derived from natural sources like mineral weathering in the crustal rocks and leaching from sediments. The anthropogenic origins of As comprise mining, smelting, usage of pesticides, herbicides, and fertilizers (Mandal and Suzuki 2002; Mahimairaja et al. 2005; Bhattacharya et al. 2021). In human beings, the main routes for As exposures are drinking water and food (Kapaj et al. 2006; Azizur Rahman et al. 2008; Santra et al. 2013).

Arsenic concentration in uncontaminated soils ranges from 1 to 40 mg/kg, with the highest and lowest concentrations observed in sandy and fine-grained alluvial and organic soils, respectively (Mandal and Suzuki 2002). Increased demand for food production has led to unregulated irrigation with As-containing groundwater in many countries globally (Roychowdhury 2008). The As threshold for drinking water is 10 μg/L (WHO 2001), while the As threshold for irrigation water is 100 μg/L (FAO 1994); nonetheless, irrigation in Hungary is carried out with groundwater containing geogenic As concentrations of up to 220 μg/L (Sandil et al. 2021). Irrigation with groundwater loaded with As causes the accretion of As in the soil. Plants, when cultivated in As-rich soil and/or irrigated with As-containing water, uptake excessive amounts of As and deposit them in their tissues, leading to elevated concentrations of As in food crops (Roychowdhury 2008; Brammer and Ravenscroft 2009; Bhattacharya et al. 2021). The build-up of such high concentrations of As in the plant’s edible part could seriously jeopardize the health of the consumer (Azizur Rahman et al. 2008). Although the majority of studies have investigated As intake from rice, a few studies have reported that vegetables contribute 10.4–25% of the daily dietary As intake (Wong et al. 2013; Ahmed et al. 2016; Ciminelli et al. 2017). Based on the dietary patterns and the amount of vegetables consumed, the following studies calculated the daily As intake values: 17.2–26 μg/day (Samal et al. 2011), 14.15 μg/day (Biswas et al. 2019), and 7.2 μg/kg FW (Islam et al. 2023). High accumulation of As in the plant hampers the plant’s metabolic functions, damaging its physiology and morphology, and even leading to plant death (Stoeva et al. 2005; Caporale et al. 2013; Gusman et al. 2013; Sandil et al. 2021). The uptake of As in plants is dependent on a multitude of factors, including plant type, species, cultivar, soil As concentration, predominant As species, pH, clay content, the concentration of other elements, and dominant agronomic conditions (Azizur Rahman et al. 2008; Santra et al. 2013). Arsenic toxicity in plants is also influenced by the developmental stage of the plant (germination, seedling development, and vegetative growth) (Liu et al. 2005). The growth stage of the plant influences its ability to uptake, translocate, and accumulate As; seedlings/young plants have been documented to have higher nutrients and contaminants uptake rate than mature plants (Gonzaga et al. 2007; Souri et al. 2019; Yang et al. 2020). In contrast, Uroic et al. (2012) reported that cucumber plants exposed to As before the flowering stage had a lower sap flow than plants exposed to As after flowering. Young plants were more efficient in restricting As loading into the xylem at high concentrations of As (0–1000 μg/kg). Stoeva et al. (2005) stated that in the early growth stages of bean, As stimulated the peroxidase activity to tackle As stress in the later stages and inhibited photosynthesis at a lower rate indicating that plants’ response to As varies with the growth stage. The plant age also influences the transfer factor (TF), wherein the TF for As in *Pteris* was 3.2, 2.1, 1.6, and 1.6 for plants aged 2, 4, 10, and 16 months, respectively. The young plants were very efficient in translocating As, and the TF reduced with age (Gonzaga et al. 2007).

In the past decade, several studies have concentrated on the uptake and accumulation of As in the mature/fruiting stage of plants, particularly in the plants’ edible part. However, scarce information is available on the uptake and translocation of As at different developmental stages in plants, which leads to a lack of understanding of As translocation from soil to plant. Only a few recent studies (Chowdhury et al. 2018; Shi et al. 2019) have reported the As uptake and allocation in plant parts in rice and wheat at different growth stages. It is essential to identify the pattern of As mobility and accumulation in the plants at different stages of growth to propose remedial measures for limiting As concentration in the edible parts. The current study examines the variations in As concentrations in the plant parts of bean and lettuce at different growth stages, and it is the first step in understanding the dynamics of As uptake, accumulation, and effect in the two investigated vegetables. The aims of our study were to 1) elucidate the impact of As treatment on the growth of plants at each developmental stage, 2) determine the difference in As translocation and accumulation among the different plant parts at each developmental stage, 3) document the difference in As uptake mechanism of fruit and a leaf vegetable, and 4) assess the potential health risks associated with the consumption of the two vegetables. Bean and lettuce were chosen for this experiment because they represent two different vegetable types (fruit and leaf), are easy to cultivate, and have a short growth duration. Bean, an As-sensitive plant, provides an economical source of protein and is cultivated as a staple crop in many countries (Caporale et al. 2013). Lettuce, a fiber-rich important leafy vegetable, is also a rich source of fiber and vitamin C (Gusman et al. 2013).

## Materials and methods

### Soil analysis

Uncontaminated soil (0–30 cm) was collected from an agricultural field in Őrbottyán, Hungary (47° 40′ N, 19° 14′ E). The bulk soil samples collected by the composite soil sampling method were air-dried, mixed thoroughly, sieved, and stored in polyethylene bags until analysis. The soil grain size was determined by the laser diffraction method (Makó et al. 2019). The soil chemical parameters, including pH, organic matter (OM), CaCO_3_, cation exchange capacity (CEC), total N, NH_4_-N, NO_3_-N, P, and K, as well as water-soluble As and pseudo-total As concentration, were measured in accordance with Sandil et al. 2019. The analytical procedures are described in Table S1. The pseudo-total and water-soluble As were quantified with an inductively coupled plasma mass spectrometer (ICP-MS; Analytik Jena, Germany) (Sandil et al. 2021).

### Plant material selection and experimental design

The germination of bean (*Phaseolus vulgaris* L. var. Golden goal) and lettuce (*Lactuca sativa* L. var. ‘Május királya’) seeds was aided by placing the seeds in the dark on Petri dishes padded with moist filter paper. Bean seeds took 2–3 days to germinate, while lettuce seeds did so in 4–5 days. The germinated seedlings were transferred to cylindrical, transparent plastic containers (0.87 L/1000 g) with soil and cultivated in a controlled growth chamber (day/night temperatures of 25–27 °C/17 °C and 16 h of lighting at a photon flux density of 500 μmol/m^2^/s) (Dobosy et al. 2020). The pots were weighed and supplied with a constant volume of irrigation water in order to preserve soil moisture at 60% of field capacity (Dobosy et al. 2020). The bottom of the pots was pierced to allow aeration and flow of leachate.

Plants were irrigated thrice weekly with uncontaminated (devoid of added As) standing drinking water. From the third week of the plantation, Hoagland’s solution and sodium arsenate (Na_2_HAsO_4_.7H_2_O) solution at concentrations 0.1, 0.25, and 0.5 mg/L, were added to the irrigation water. The range of As concentrations were selected to incorporate the As concentrations frequently encountered in groundwater in As-afflicted regions around the globe (Sarkar and Paul 2016; Smedley 2018). A full factorial random experimental design was adopted using 5 replicates for all combinations of As treatments (4-level factor) and developmental stages (2- and 3-level factor for lettuce and bean, respectively) (Table S2). A set of control plants irrigated with uncontaminated water were cultivated alongside. Bean plants were harvested at young (2–3 leaflets), flowering, and fruiting (2–3-inch-long pods) stages, while lettuce plants were harvested at young (7–8 leaves) and mature (head development) stages.

### Plant harvest and sample preparation and analysis

Harvested plants were separated into root, stem, leaves, and flower/fruit in the case of bean and root and leaf in the case of lettuce. The preparation of the sample and the elemental analyses were carried out in accordance with Sandil et al. 2021. Deionized water was used to clean the plant samples, and their fresh weights (FW) were recorded. The samples were then dried in a laboratory dryer at 40 °C for 48 h to achieve a constant dry weight (DW) and the dried samples were homogenized with an agate pestle and mortar. The total As concentration in samples was ascertained by digesting 0.1–0.5 g of sample with HNO_3_ and H_2_O_2_ (7 mL : 3 mL) in a microwave-assisted acidic digestion system. ICP-MS (Analytik Jena, Germany) was used to quantify the total As concentration in the samples. The accuracy of the As measurement was verified using certified reference material (NIST 1573a tomato leaf-National Institute of Standards and Technology) (Sandil et al. 2021).

### Transfer factor (TF)

The As transfer factor (TF) was determined according to Sandil et al. (2021):

1 $$\mathrm{TF}=\left[\mathrm{As}\ \mathrm{concentration}\ \mathrm{in}\ \mathrm{edible}\ \mathrm{part}\right]/\left[\mathrm{As}\ \mathrm{concentration}\ \mathrm{in}\ \mathrm{root}\right]\kern8em$$

### Daily dietary As exposure

The estimated daily intake (EDI) for As was appraised according to Sharma et al. (2016):

2 $$\mathrm{EDI}=\left[\mathrm{C}\times \mathrm{IR}\times \mathrm{Cf}\right]/\left[\mathrm{BW}\right]$$

where C, IR, Cf, and BW stand for the As concentration in the edible part of vegetables (mg/kg DW), daily vegetable consumption rate, conversion factor from FW to DW, and average body weight, respectively (Sandil et al. 2021). Table S3 lists the IR, Cf, and BW values for adults and children.

### Health risk assessment

The hazard quotient (HQ), an estimate of the potential non-cancerous health effects, was derived based on Rehman et al. (2016):

3 $$\mathrm{HQ}=\left[\mathrm{EDI}\right]/\left[\mathrm{RfD}\right]$$

where EDI is as defined above; RfD is the oral reference dose of As (3×10^-4^ mg/kg As per day), daily exposure to which will not result in any adverse effect over the course of a lifetime (USEPA 2012; Ramirez-Andreotta et al. 2013).

The estimated daily exposure (EDE) to As was calculated in accordance with Rehman et al. (2016):

4 $$\mathrm{ED}\mathrm{E}=\left[\mathrm{C}\times \mathrm{IR}\times \mathrm{Cf}\times \mathrm{EF}\times \mathrm{ED}\right]/\left[\mathrm{BW}\times \mathrm{LE}\right]$$

where EF, ED, and LE are the exposure frequency, exposure duration, and life expectancy, respectively.

The carcinogenic hazard, determined by the lifetime cancer risk (LCR), was estimated based on the modified formula of Ramirez-Andreotta et al. (2013):

5 $$\mathrm{LCR}=\left[\mathrm{EDE}\right]\times \left[\mathrm{CSF}\right]$$

where CSF is the cancer slope factor. The values of RfD, EF, ED, LE, and CSF are enumerated in Table S3.

### Statistical analysis

Statistical analysis was carried out using the R statistical software (R Core Team 2019). The effects of various treatment dosages on the plant As concentration and dry mass were compared using linear regression models. The “glht” function of the “multcomp” package (Hothorn et al. 2008) was used to perform post hoc pairwise comparisons using Tukey multiple comparisons of means (Sandil et al. 2021). Comparisons where the statistical tests resulted in a *p*-value of less than 0.05 were deemed significant in all cases. Figures were prepared in R and Microsoft Excel 2013 (Microsoft Corp. USA).

## Results and discussion

### Soil parameters

Table S4 lists the physical and chemical characteristics of the soil. These soil parameters, including soil particle size, texture, OM, pH, redox potential, presence of elements (Fe, P, S, Al, and CaCO_3_), and mineral nutrients content, regulate the soil As concentration, mobility, availability, and toxicity of As (Azizur Rahman et al. 2008; Brammer and Ravenscroft 2009; Sandil et al. 2021). Additionally, the uptake and metabolism of As are influenced by a number of microorganisms (Kabiraj et al. 2020; Kabiraj et al. 2022). The soil was categorized as calcareous sandy soil based on the high fraction of sand and CaCO_3_ content. Sandy soils are characterized by a reduced capacity for As adsorption compared to clayey soils because they contain a lower amount of clay, OM, and oxides of Fe and Al, which ensures a higher As mobility and bio-availability in such soils. But calcareous soils can have higher levels of As than non-calcareous soils (Mahimairaja et al. 2005; Azizur Rahman et al. 2008). The pH was alkaline, probably as a consequence of calcium carbonate, and the CEC appeared to be dependent on the low OM content and clay fraction.

The pseudo-total As concentration in the soil was 3.50 mg/kg, though just a dismal 0.66% of it was water-soluble (Sandil et al. 2019), presumably a result of elevated Fe (8420 mg/kg) and Ca (16.1 w/w%) concentration in the soil. Ferrous oxides and hydroxides are ordinarily involved in regulating As availability in soils due to their high adsorption affinity for As (Mahimairaja et al. 2005) and in soil layers with an abundance of Fe, As precipitates as ferric arsenate (Mandal and Suzuki 2002). Similarly, Ca in the soil form Ca-As precipitates, which are less soluble and reduce the release of As from the soil (Long et al. 2023). However, considering the soil’s high P content (129 mg/kg), the As bio-availability could be higher. In soil, P and As ions contend for common sorption sites, and P has been reported to increase As solubility by displacing As from the binding sites (Mahimairaja et al. 2005).

### Effect of arsenic on plant growth at different developmental stages

In lettuce, the root and leaves biomass production were measured at the plant’s young and mature growth stages (Fig. S1). In lettuce, there were no visible signs of As toxicity at any As treatment or growth stage. The roots displayed a similar trend at both growth stages; at As treatments of 0.1 and 0.5 mg/L, they exhibited a negative biomass output relative to the control, while at 0.25 mg/L, they showed an increment in growth. In contrast, the lettuce leaves grew uninhibited at all As treatments in both growth stages. However, the changes in the biomass of roots and leaves were not significant at either growth stage and any As treatment. Koo et al. (2011) reported comparable observations in lettuce; they noted the unhindered growth of shoots at all As treatments and the existence of significant negative correlations (*r* < − 0.70) between root growth and As concentration, implying higher sensitivity of lettuce roots. In contrast, Gusman et al. (2013) documented that the lettuce leaf biomass continuously declined as the As concentration increased (0–4 mg/L), without any visual toxicity symptoms. The root biomass initially increased at a low As concentration (0.5 mg/L) and then declined as the As concentration enhanced (1–4 mg/L). The authors reported the initial increase in root biomass to be related to P nutrition; when exposed to low concentrations of As, plants uptake higher amounts of P, increase their photosynthetic rate, and display an increase in biomass. The higher P uptake is enabled by P deficiency in the plant since As replaces P in several metabolic pathways, but cannot carry out its functions.

In beans, the growth was analyzed at three stages, young, flowering, and fruiting (Fig. S2). No visible phytotoxicity symptoms were observed at any growth stage in the bean plant. At the young and flowering stages of the plant, the growth of all plant parts was affected by the increase in As concentration. The As treatment root biomasses were higher than the control at all growth stages. However, the roots at the young and flowering stage displayed a reduction in biomass with rising As concentration. This could be due to the higher sensitivity of younger plant tissues (Miteva 2002). Contrarily, the roots at the fruiting stage displayed positive growth, increasing with the As concentration applied. In the stems, the As treatment of 0.25 mg/L caused an aberration in growth at all developmental stages. The relative stem biomasses were the following: young stage (8.30%, − 32.87%, and 7.01%), flowering stage (− 17.94%, − 27.96%, and − 21.44%), fruiting stage (10.68%, 14.56%, and 3.46%) at As treatment of 0.1, 0.25, and 0.5 mg/L, respectively. At the flowering stage, the stem biomass reduced relative to the control at all As treatments, while in the fruiting stage, it increased varyingly. The As accumulation in the stem was extremely high in the flowering stage (Fig. 2b) compared to the other two growth stages, which could have inhibited the stem biomass. In the leaves, positive relative growth was observed at all stages, except at 0.1 mg/L As treatment in the flowering stage. We observed an overall reduced stem growth and erratic behavior in the leaf biomasses at the flowering stage of the plant, which could be because of the redistribution of essential elements and As in the plant parts in preparation for fruit development. The bean plant at the fruiting stage displayed an increment in the growth of all plant parts except the fruit. The bean fruit appeared extremely susceptible to elevated As concentrations, perhaps as a result of increased As accumulation in the vegetative parts, which could affect the fruit development. On the other hand, the roots remained unaffected by As exposure, possibly because As in plants is sequestered mainly in the roots by complexation with phytochelatins and sequestration in root vacuoles (Yañez et al. 2019).

Similarly, no change in the root and shoot biomasses was noted in the castor bean plant (*Ricinus communis* cv. Guarany) at As concentrations of 0.01–0.5 mg/L. The authors recorded an insignificant decrease of 23% in the root biomass and a significant decrease of 35% in the shoot biomass at a much higher As dosage (5 mg/L), but without any observable toxicity symptoms (Melo et al. 2009). On the contrary, Caporale et al. (2013) noticed that as the As treatment increased (0–3 mg/L), the bean (*Phaseolus vulgaris* L.) biomass decreased, and phytotoxicity symptoms started to manifest (leaves with reddish-brown necrotic patches). Carbonell-Barrachina et al. (1997) also reported a comparable decline in bean biomass at As dosages of 2 and 5 mg/L.

### Arsenic accumulation in plants

**Fig. 1:**
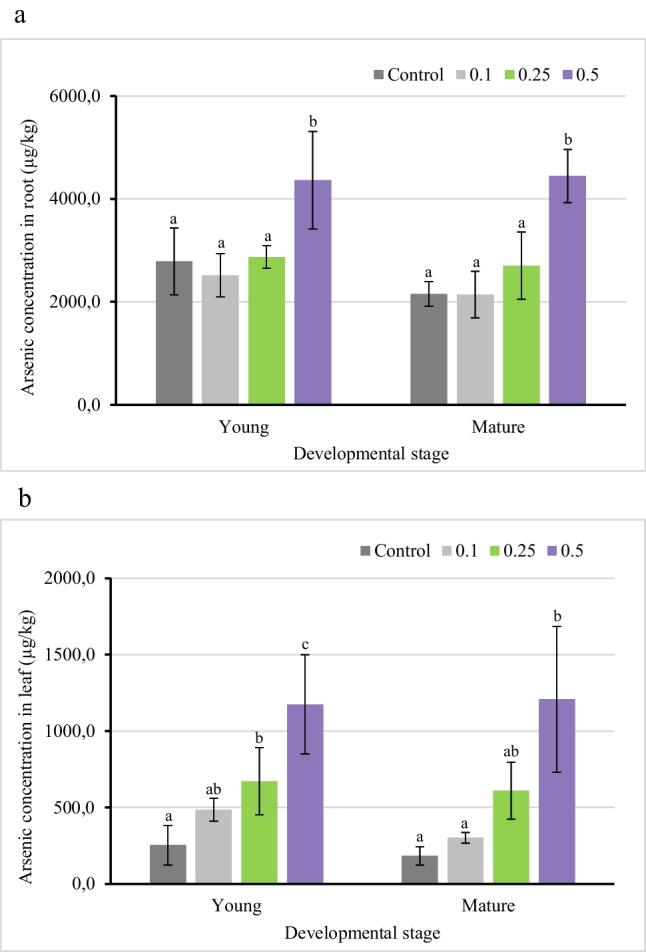
Arsenic concentration in lettuce plant parts (**a**. root, and **b**. leaves) at different growth stages when irrigated with water containing As in concentrations of 0, 0.1, 0.25, and 0.5 mg/L. Error bars indicate standard deviation (*n*=5). Different letters indicate significant differences among treatments (*p*<0.05)

The increase in the As concentration of irrigation water resulted in an increase in As concentration of both root and leaves of lettuce (Fig. 1). At both growth stages, lettuce roots accumulated high concentrations of As; however, a significant increase in the roots As concentration at both growth stages was observed solely with the highest As treatment (0.5 mg/L). The plants exposed to lower As concentrations differed neither from the control nor from each other. The leaves, in comparison to the roots, contained a lower amount of As. In lettuce leaves at the young stage, the As accumulation increased with increasing As concentration in the irrigation water, but was significantly different only at 0.25 and 0.5 mg/L As treatment. At the mature stage, the leaves accumulated significantly higher As concentration only at the highest As treatment. At both the growth stages of lettuce, the As accumulation in roots and leaves at 0.5 mg/L As treatment was approximately twice higher as compared to 0.25 mg/L treatment. Considering the plant biomass at both stages, it is evident that lettuce at the young stage accumulated a higher concentration of As.

In lettuce, Gusman et al. (2013) also witnessed the accumulation of a higher amount of As in the roots and leaves upon exposure to increasing As concentrations. In their study, an increase in the applied As treatment (0.5–4 mg/L) caused a corresponding increase in the As concentration of leaves (24.64–34.94 mg/kg DW) and roots (245.7–319.7 mg/kg DW). In lettuce cultivated in control soil and distilled water, Yañez et al. (2019) reported the As accumulation to be 7.12 mg/kg DW in root and below detection in leaves. But when the distilled water was replaced with water containing As (1.44 mg/L), the As accumulation in root and leaves increased to 40.5 and 8.76 mg/kg DW, respectively.

**Fig. 2:**
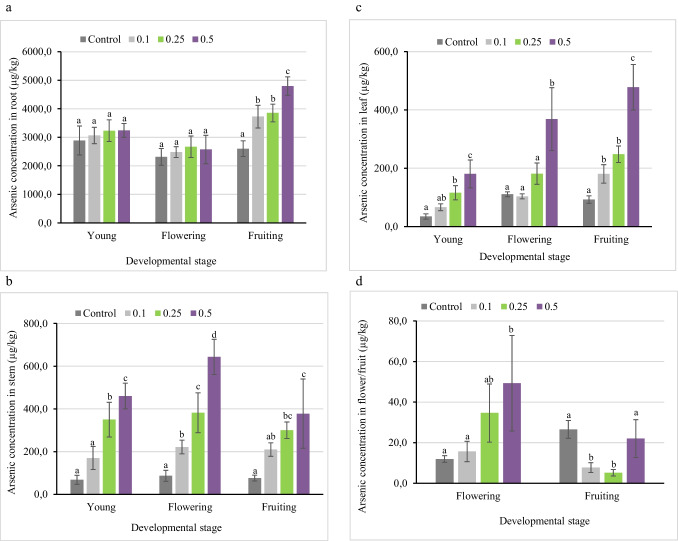
Arsenic concentration in bean plant parts (**a**. root, **b**. stem, **c**. leaves, and **d**. flower/fruit) at different growth stages when irrigated with water containing As in concentrations of 0, 0.1, 0.25, and 0.5 mg/L. Error bars indicate standard deviation (*n*=5). Different letters indicate significant differences among treatments (*p*<0.05)

In bean, the increase in As concentration in the irrigation water resulted in increased As concentration of all plant parts, except the fruit (Fig. 2). The roots, at all growth stages, accumulated the highest As concentration. The increase in As concentration of roots with increasing As treatment was not significant at the young and the flowering stage, but at the fruiting stage, the roots accumulated significantly higher As concentrations even on the application of the lowest As concentration (0.1 mg/L). The roots of the flowering plant were found to contain lesser As concentration than the young and fruiting plant roots, probably due to higher translocation of the element to the shoots. The root activity of plants has been reported to reduce significantly during the flowering stage, and it could also be considerably reduced in the fruiting and seed-setting stage (Souri et al. 2019). The As concentration of roots compared to the respective biomass was highest at the young growth stage.

The increase in As concentration in the irrigation water resulted in a significant increase in As concentration of the stem and leaves of bean plants at all growth stages. The plant stem at the flowering stage contained a greater As concentration than the young and fruiting plant’s stems due to increased translocation and uptake of nutrients to prepare for the development of the fruits. The As concentration in the leaves increased with the growth stage, with the fruiting stage containing the highest As concentration. At 0.1, 0.25, and 0.5 mg/L As treatment, the bean flowers contained 16, 35, and 49 μg/kg DW As concentrations, respectively, and at the same treatments, the bean fruit contained the lowest As concentrations (8, 5, and 22 μg/kg DW). Carbonell-Barrachina et al. (1997) reported that bean root and fruit contained As concentrations of 30.4 and 43 mg/kg DW and 4.4 and 3.3 mg/kg DW, respectively, at As treatment of 2 and 5 mg/L. They noted that an elevation in the As concentration resulted in a decline in the amount of As translocated, suggesting the presence of a restriction in the As pathway in bean. The restriction was likely due to root cell damage caused by high concentrations of As.

In both bean and lettuce, an elevation in the As concentration of irrigation water induced a rise in the As concentration in all plant parts at all growth stages, with the roots and the terminating point of the phloem distribution path, respectively, containing the highest and lowest As concentration. This implies that roots are effective deterrents against As transfer to the plant’s aboveground components. Other studies have noted the same trend in bean (Carbonell-Barrachina et al. 1997; Caporale et al. 2013; Yañez et al. 2019) and lettuce (Gusman et al. 2013; Yañez et al. 2019). Roots contain a higher As concentration because they are in direct contact with the contaminated medium and secrete a range of metabolites to enhance As uptake; thus, ab-/adsorption is the highest in these tissues. Furthermore, plants form As-PCs (As-phytochelatin) complexes and sequester them in the root vacuoles to reduce the As translocation to above-ground parts. Since roots, as opposed to shoots, do not expand considerably in length and width, the As concentration sequestered in the roots is not diluted. Plants also prevent As from reaching photosynthetic tissues by reducing sap flow and limiting translocation (Carbonell-Barrachina et al. 1997; Chowdhury et al. 2018; Souri et al. 2019). The concentration of As in the roots of both plants determined the amount of As present in the edible part. Lettuce contained a higher As concentration in the edible part than bean, probably due to its large leaf area, short translocation pathway, and enhanced transpiration rate. Leafy vegetables have been reported to accumulate a higher amount of As than non-leafy vegetables (Huang et al. 2006). The amount of As absorbed by plants varies with the plant type, growth stage, habitat, and root morphology (length, diameter, and root hair) (Abedin et al. 2002; Rofkar and Dwyer 2011).

### Total arsenic concentration in plants at the various growth stages

**Fig. 3:**
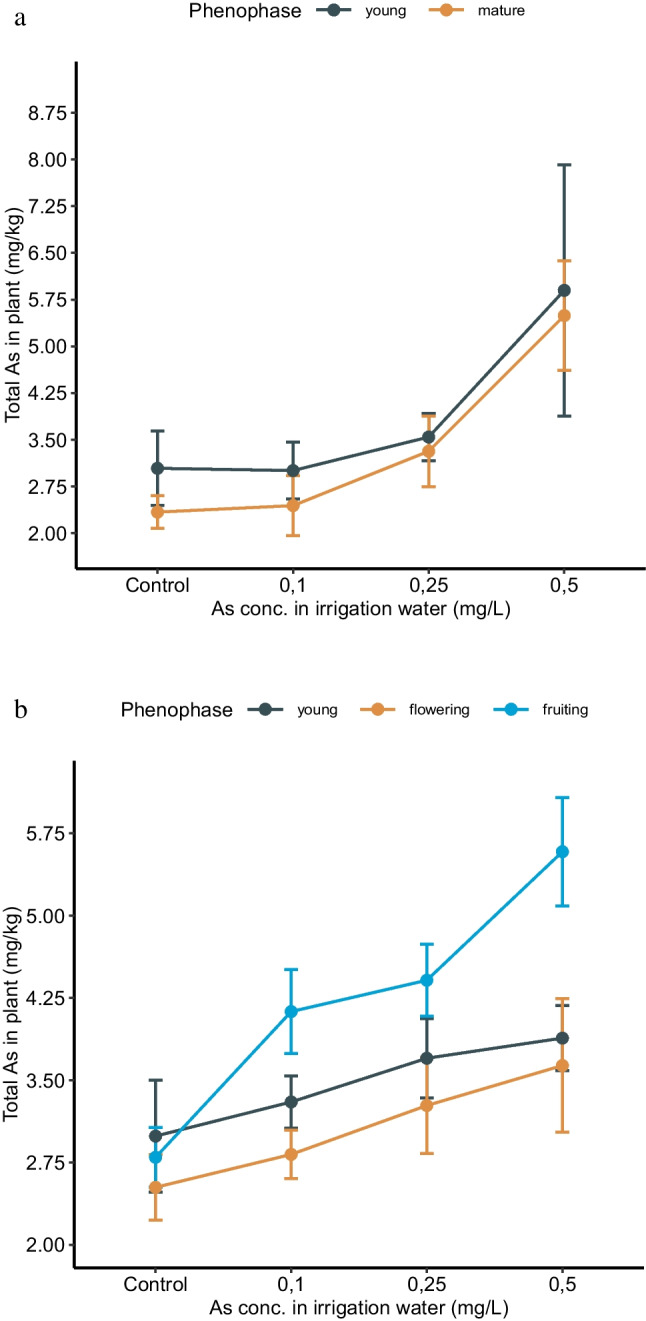
Arsenic concentration in the (**a**) entire lettuce plant (root+leaves) and (**b**) entire bean plant (root+stem+leaves+flower/fruit) at different growth stages when irrigated with water containing As in concentrations of 0, 0.1, 0.25, and 0.5 mg/L. Error bars indicate standard deviation (*n*=5)

Based on the biomasses of both plants at the different growth stages observed in our study, it could be stated that bean and lettuce accumulated a greater concentration of As at the younger growth stage. The As concentration in the entire lettuce plant and the entire bean plant at the different growth stages is shown in Fig. 3.

At all the As treatment levels applied in our study, lettuce accumulated a higher concentration of As in the young growth stage compared to the mature stage. However, the As concentration at the growth stages was significantly different only in the control plant (*p*<0.05). In bean, the control plant contained the As concentration in the following order: young>fruiting>flowering stage, but the differences in As accumulation were not significant. In all the other As treatments, the As accumulation was in the order: fruiting>young>flowering stage. In these treatments, the differences in As accumulation were significant among the young-fruiting and flowering-fruiting stages (*p*<0.05).

The physiological characteristics of plants are dependent on the age of the plant and affect the biomass output and uptake and accretion of nutrients and contaminants. A plant’s ability to accumulate As is controlled by its roots. Roots, at the various stages of growth, exhibit diverse nutrient absorption behavior, depending upon the requirement of the plant. Compared to older roots, young roots tend to absorb nutrients at a greater rate due to their augmented growth activity, and as plants age, their average absorption rate per unit of root declines (Gonzaga et al. 2007; Rofkar and Dwyer 2011). In rice, Chowdhury et al. (2018) noted a similar As accumulation trend, with the highest As concentration occurring during the vegetative phase, succeeded by an acute decline in As concentration during the reproductive phase and an increase in As during the grain ripening phase. In tomato plants exposed to excessive As in soil, the As accumulation in the primary leaf was rigorous and showed a strong correlation with the administered As doses (Miteva 2002). The plant’s reaction to As during the early stages of growth denotes the sensitivity of young plant tissues and their capacity to amass a greater concentration of As compared to older plants (Miteva 2002). Gonzaga et al. (2007) also noted a decline in the As accumulation potential of *P. vittata* as the plant aged. In comparison to plants aged 4 and 16 months, the As accumulation in the fronds of a 2-month-old plant was 36% higher. The As partitioning also depended on the age of the plant; in the 2-month-old plant, 85% As was in the leaf and 15% in the root, while in the 10-month-old plant, 67% As was in the leaf and 33% in the root (Gonzaga et al. 2007).

### Transfer factor (TF) at different developmental stages

The transfer factor (TF) (Table 1) signifies a plant’s efficacy in moving As from the root to the shoot (Singh and Ma 2007). Plants that are not hyperaccumulators of As generally have a TF value lower than 1 (Singh and Ma 2007). The overall root-to-shoot TF ranged from 0.08 to 0.27 in lettuce and 0.04 to 0.41 in bean. The root-to-flower/fruit TF in bean in our results as well as in literature data (Huang et al. 2006; Bergqvist et al. 2014) is very low. The low TFs are possibly the plant’s tool to thwart the loss of photosynthetic tissues and diminish As phytotoxicity (Melo et al. 2009; Bergqvist et al. 2014). Many plants exhibit poor soil-to-plant As translocation due to low bioavailable As in soil, constrained absorption by roots, restricted transfer from root to shoot, and phytotoxicity of As at even low concentrations (Singh and Ma 2007).

**Table 1:** Transfer factor for As in bean and lettuce irrigated with different concentrations of As-containing water

	Control	0.1 mg/L	0.25 mg/L	0.5 mg/L
Bean (root to shoot)				
Young stage	0.04	0.08	0.15	0.19
Flowering stage	0.09	0.14	0.22	0.41
Fruiting stage	0.08	0.11	0.14	0.18
Bean (root to flower/fruit)				
Flowering stage	0.005	0.006	0.013	0.019
Fruiting stage	0.010	0.003	0.002	0.005
Lettuce (root to shoot)				
Young stage	0.09	0.19	0.23	0.27
Mature stage	0.08	0.14	0.23	0.27

Overall, lettuce showed a higher root-to-shoot translocation efficiency than bean at both stages of growth. This could be because i) lettuce has a shorter translocation pathway from root to leaves, facing lesser sequestration and uptake barriers, while in bean, the translocation pathway is extended, and the As is sequestered in the root, stem, and leaves, and only a small amount reaches the fruit and ii) the biomass production in lettuce root and leaves was much higher than bean, which could help in higher absorption of As. Lettuce was tolerant to all concentrations of As and grew uninhibited, accumulating more As in both plant parts.

In lettuce, the TF was higher at the young stage in the control and As treatment of 0.1 mg/L and reduced with plant age. Similarly, in *Pteris*, Gonzaga et al. (2007) noted that plants in early developmental stages were highly efficient As accumulators and displayed a decrease in the TF with increasing plant age. In plants that were 2, 4, 10, and 16 months old, the corresponding TF values were 3.2, 2.1, 1.6, and 1.6. The ability of the young plants to accumulate more As could be due to several factors: i) the concentration of glutathione, an important antioxidant and the precursor of PCs, is highest at the young stage of the plant, and decreases with plant age and ii) the ability of the root to uptake nutrients decreases with plant age, thus young plants absorb higher amounts of contaminant as compared to old plants (Gonzaga et al. 2007). Interestingly, an increase in As treatment (0.25 and 0.5 mg/L) resulted in similar TF at both growth stages. On the other hand, in beans, the highest TF was observed at the flowering stage. Rofkar and Dwyer (2011) also observed higher TF in older plants of *Carex stricta* and *Spartina pectinate*. In *Carex,* the TF increased from 0.1 in the young plant to 0.45 in the old plant, while in *Spartina,* it changed from 0.01 to 0.07. The authors reported that As translocation increased with increasing age and decreasing growth rates, but did not state any explanation for this behavior. Additionally, they noticed that the concentration of Fe and P in the plant roots increased with plant age, suggesting a role of these nutrients in the plant’s ability to concentrate and translocate As. In our study, the higher root-to-shoot TF in beans in the flowering stage could be caused by the roots having a low As concentration and the stem and leaves having a comparatively larger As concentration. The increase in applied As concentration resulted in an enhanced TF in both bean and lettuce at all growth stages. The higher TFs were probably caused by the decreasing capacity of the roots to retain the As up-taken by the plant as a result of the medium’s growing As concentration. Melo et al. (2009) observed a similar increase in the TF with increasing As concentration in castor bean.

### Health risk assessment

Arsenic exposure in human beings through plants depends on the plant type, physiology, ability to translocate As to edible parts, the quantity of vegetables consumed, and the frequency of consumption (Huang et al. 2006; Azizur Rahman et al. 2008; Santra et al. 2013). A common metric for determining the health risks associated with As intake has been the provisional tolerable daily intake (PTDI) value of 2.14 μg As per kg BW per day. But As intake at the PTDI value has now been known to result in various cancers (FAO/WHO 2010) and has thus been replaced by the EDI and HQ values, which provide a better measurement of As-associated risk.

In comparison to lettuce leaves (0.30, 0.61, and 1.21 mg/kg DW), the As accumulation in the bean fruit (0.008, 0.005, and 0.022 mg/kg DW) was substantially lower at As treatment of 0.1, 0.25, and 0.5 mg/L, respectively. Only the As values in bean at all concentrations were less than the FAO/WHO maximum acceptable limit of 0.1 mg/kg. The EDI, HQ, and LCR values are listed in Table 2.

**Table 2:** The values of estimated daily intake (EDI), hazard quotient (HQ), and lifetime cancer risk (LCR) for bean and lettuce

Plant type	Arsenic treatment (mg/L)	EDI (mg/kg/day)		HQ		LCR	
		Adult	Child	Adult	Child	Adult	Child
Bean	C	1.59×10^−5^	2.07×10^−5^	5.31×10^−2^	6.89×10^−2^	2.39×10^−5^	9.31×10^−5^
	0.1	4.65×10^−6^	6.04×10^−6^	1.55×10^−2^	2.01×10^−2^	6.98×10^−6^	2.72×10^−5^
	0.25	3.11×10^−6^	4.04×10^−6^	1.04×10^−2^	1.35×10^−2^	4.66×10^−6^	1.82×10^−5^
	0.5	1.32×10^−5^	1.72×10^−5^	4.41×10^−2^	5.73×10^−2^	1.99×10^−5^	7.74×10^−5^
Lettuce	C	1.10×10^−4^	1.42×10^−4^	3.65×10^−1^	4.74×10^−1^	1.64×10^−4^	6.40×10^−4^
	0.1	1.81×10^−4^	2.35×10^−4^	6.02×10^−1^	7.83×10^−1^	2.71×10^−4^	1.06×10^−3^
	0.25	3.65×10^−4^	4.75×10^−4^	1.22×10^0^	1.58×10^0^	5.48×10^−4^	2.14×10^−3^
	0.5	7.25×10^−4^	9.42×10^−4^	2.42×10^0^	3.14×10^0^	1.09×10^−3^	4.24×10^−3^

For beans, at all As concentrations, the EDI and HQ values were below 1, and the EDI was also below the RfD value of 3×10^−4^ mg/kg As per day, implying no significant health risk from the consumption of bean. Rehman et al. (2016) observed similar EDI and HQ values of less than 1 in vegetables from an agricultural field (soil As: 3–3.9 mg/kg) where the vegetables accumulated 0.03–1.38 mg/kg As concentration. In lettuce, due to higher As accumulation, the EDI values for adults and children at 0.25 and 0.5 mg/L As treatment were above the RfD value, implying significant health hazards. This higher EDI also translated into an HQ value above 1, signifying that lettuce consumption at these concentrations would cause potential non-cancerous risks. The EDI and HQ scores for children were markedly greater due to their smaller bodies and high As burden (Roychowdhury 2008). The LCR (carcinogenic risk) with an acceptable range between 1 in 10,000 to 1 in 1,000,000 denotes the excess cancer risk for an individual (beyond the already existing risk of acquiring cancer) when they consume vegetables in accordance with the guidelines used to calculate the EDE (USEPA 2012). Consumption of bean cultivated at any As concentration did not pose a carcinogenic risk, but lettuce cultivated at all As treatments could cause lifetime cancer risk in adults and children, with the cancer risk being very high in children. But it is possible that the risks could be overstated in our study because the calculations are based on the total As and not on the inorganic As concentration.

## Conclusion

In beans, the decrease in biomass production of fruit became more pronounced with increasing As treatment. Considering the health risk assessment values obtained in our study, the consumption of bean irrigated with As concentration up to 0.5 mg/L is acceptable. On the contrary, in lettuce, the biomass production of leaves was enhanced on irrigation with a higher As concentration. But for health risk-free consumption of lettuce, it should be ensured that the irrigation water used is entirely free of As. Considering the biomass production and health risks in both cases, it is recommended for farmers that both these plants should be cultivated with As-free water and in uncontaminated/minimal As-containing soil. But this recommendation is valid only for calcareous sandy soil and the As concentrations applied in this study. Further investigations would be needed to provide an informed commendation, preferably in field conditions with a broader range of As concentrations and soil types.

### Supplementary information

ESM 1 (DOCX 440 kb)

## Data Availability

The data supporting the findings of this study are available within the article and its supplementary materials.

## Abbreviations

EDI: Estimated daily intake
EDE: Estimated daily exposure
FAO: Food and Agriculture Organization
HQ: Hazard quotient
IARC: International Agency for Research on Cancer
LCR: Lifetime cancer risk
NIST: National Institute of Standards and Technology
PTDI: Provisional tolerable daily intake
TF: Transfer factor
USEPA: United States Environmental Protection Agency
WHO: World Health Organization
