# Supplementary material for: Arsenic uptake and accumulation in bean and lettuce plants at different developmental stages
Source: Environ Sci Pollut Res Int. 2023 Nov 2;30(56):118724–35. doi: 10.1007/s11356-023-30593-7 (PMC10697903; doi:10.1007/s11356-023-30593-7)
Supplement: Supplementary file 1 — (DOCX 440 kb) [file 11356_2023_30593_MOESM1_ESM.docx]

**Supplementary information**

**Arsenic uptake and accumulation in bean and lettuce plants at different developmental stages**

Sirat Sandil^1,2^, Anett Endrédi^2^, Anna Füzy^3^, Tünde Takács^3^, Mihály Óvári^4^, Gyula Záray^1,2^, Péter Dobosy^2^*****

^1^Cooperative Research Centre of Environmental Sciences, Eötvös Loránd University, Pázmány Péter sétány 1/A, H-1117 Budapest, Hungary; [sirat.sandil@ecolres.hu](mailto:sirat.sandil@ecolres.hucom); [zaray.gyula@ecolres.hu](mailto:zaray.gyula@ecolres.hu)

^2^Institute of Aquatic Ecology, HUN-REN Centre for Ecological Research, Karolina út 29-31, H-1113 Budapest, Hungary; [endredi.anett@ecolres.hu](mailto:endredi.anett@ecolres.hu); [dobosy.peter@ecolres.hu](mailto:dobosy.peter@ecolres.hu)

^3^Institute of Soil Sciences, HUN-REN Centre for Agricultural Research, Herman Ottó út 15, H-1022 Budapest, Hungary; fuzy.anna@atk.hu, [takacs.tunde@atk.hu](mailto:takacs.tunde@atk.hu)

^4^Nuclear Security Department, HUN-REN Centre for Energy Research, Konkoly-Thege Miklós út 29-33, H-1121 Budapest, Hungary; ovari.mihaly@ek-cer.hu

*Corresponding Author: Dr. Péter Dobosy

Email: [dobosy.peter@ecolres.hu](mailto:dobosy.peter@ecolres.hu)

**Supplementary Figures**

**S1.a**
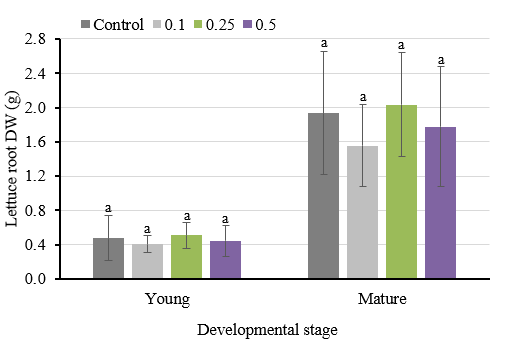


**S1.b**
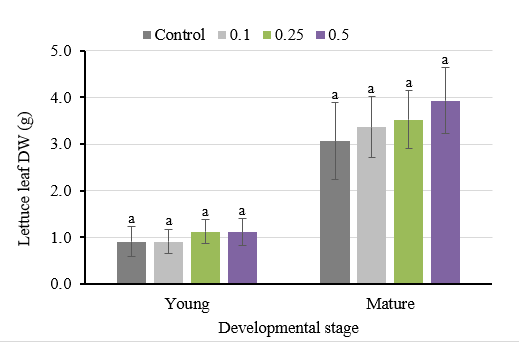


**Fig. S1** Effect of various arsenic concentrations in the irrigation water on biomass production of lettuce (a) root and (b) leaves at the two growth stages. Error bars indicate standard deviation (n=5). Different letters indicate significant differences among treatments (p<0.05)

**S2.a**
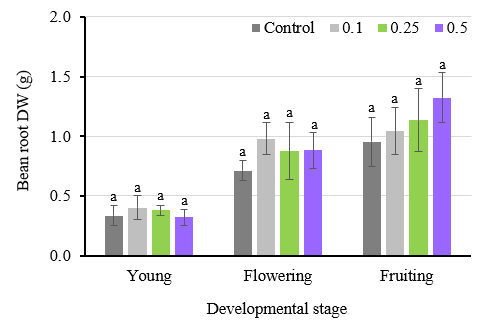


**S2.b**
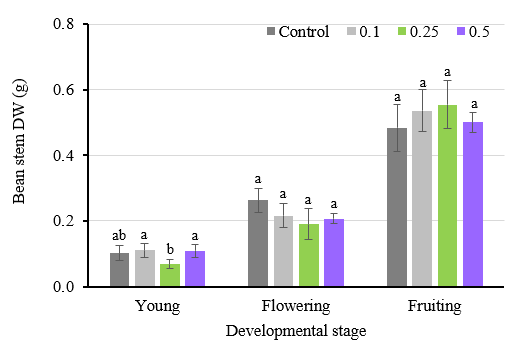


**S2.c**
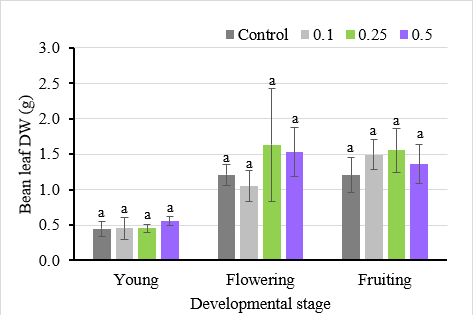


**S2.d**
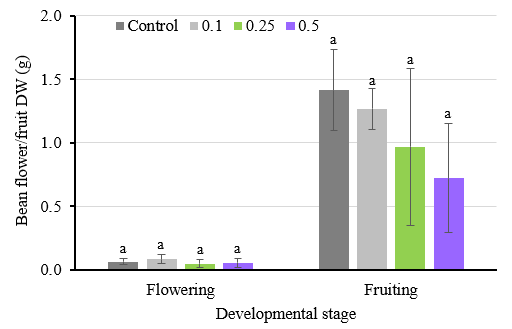


**Fig. S2** Effect of various arsenic concentrations in the irrigation water on biomass production of bean (a) root, (b) stem, (c) leaves, and (d) flower/fruit at the various developmental stages. Error bars indicate standard deviation (n=5). Different letters indicate significant differences among treatments (p<0.05)

**Supplementary Tables**

| **Soil parameter** | **Analytical method** |
| --- | --- |
| pH | Soil-water solution (1:2.5) after mixing for 12 hours (MSZ-08-0206/2:1978) |
| Organic matter | Modified Walkley-Black method (MSZ-08-0452:1980) |
| Cation exchange capacity (CEC) | Modified Mehlich method (MSZ-08-0215:1978) |
| CaCO_3_ | Scheibler gas-volumetric method (MSZ-08-0206/2:1978) |
| P and K | Extraction with ammonium-acetate lactate (Egnér et al., 1960) |
| Total nitrogen | Kjeldahl method (ISO 11261:1995) |
| NH_4_-N and NO_3_-N | KCl extracts (MSZ 20135:1999) |
| Pseudo-total As | Aqua-regia digestion (MSZ 21470-50:2006) |
| Water-soluble As | Soil-water solution (1:10) |

**Table S1** Analytical methods used for the chemical characterization of soil (Sandil et al. 2019)

|  | | **Plant developmental stages** | | | | |
| --- | --- | --- | --- | --- | --- | --- |
|  |  | Lettuce (2 level factor) | | Bean (3 level factor) | | |
|  |  | Young | Mature | Young | Flowering | Fruiting |
| **Arsenic treatment** (4 level factor) | 0 mg/L | 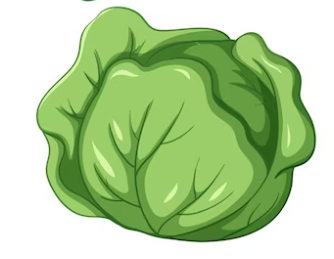  n = 5 | 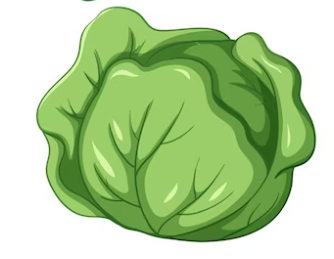  n = 5 | 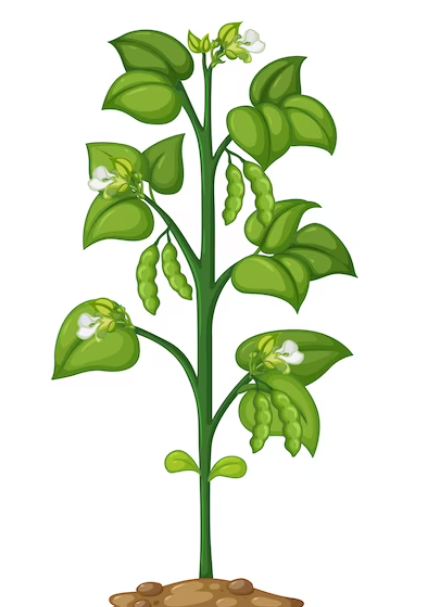  n = 5 | 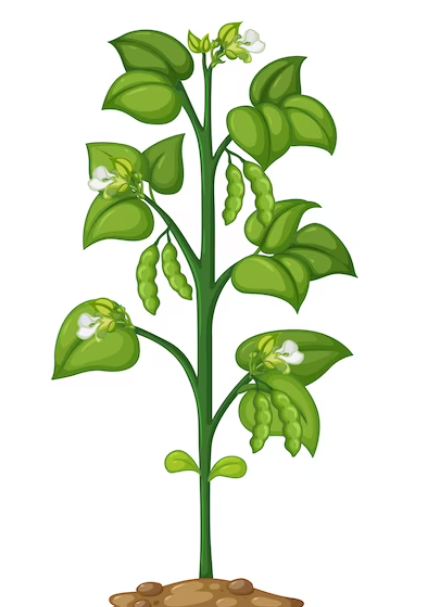  n = 5 | 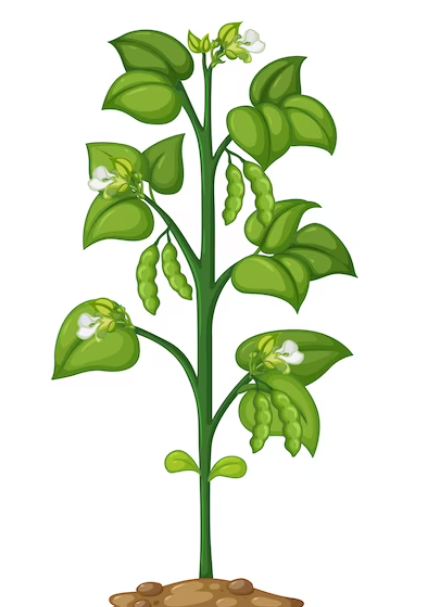  n = 5 |
|  | 0.1 mg/L | 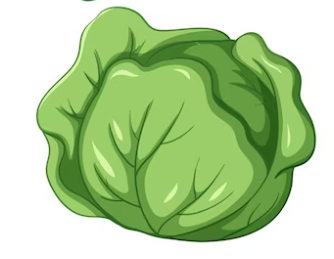  n = 5 | 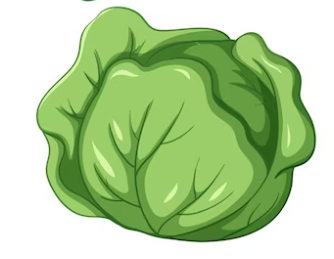  n = 5 | 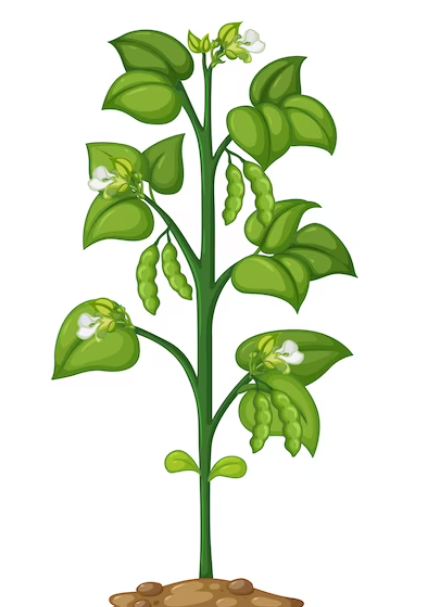  n = 5 | 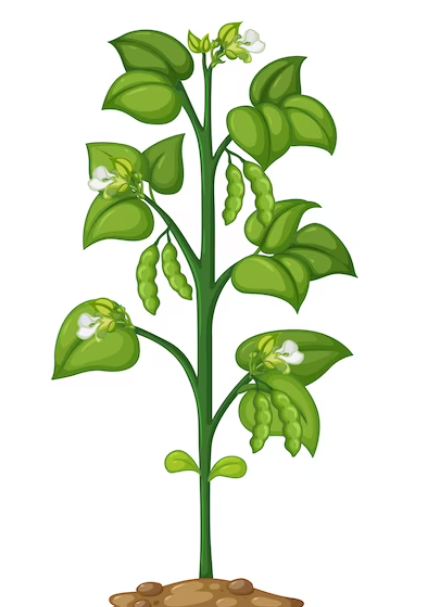  n = 5 | 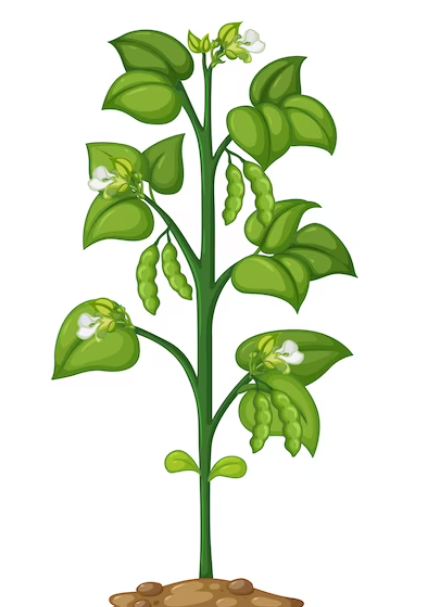  n = 5 |
|  | 0.25 mg/L | 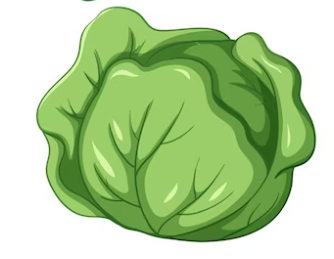  n = 5 | 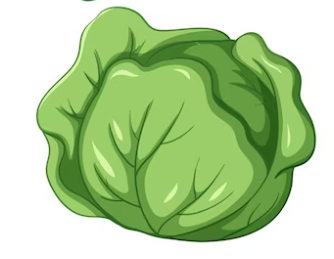  n = 5 | 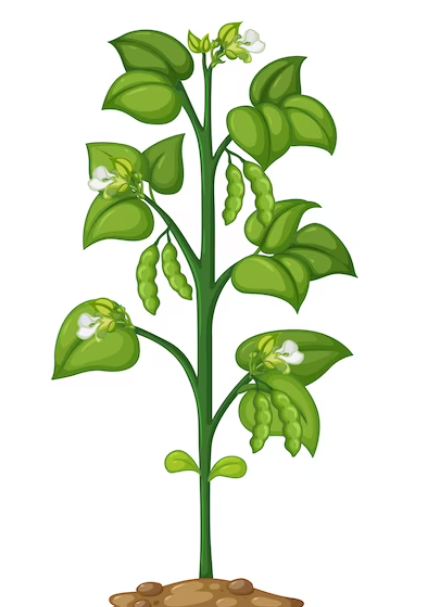  n = 5 | 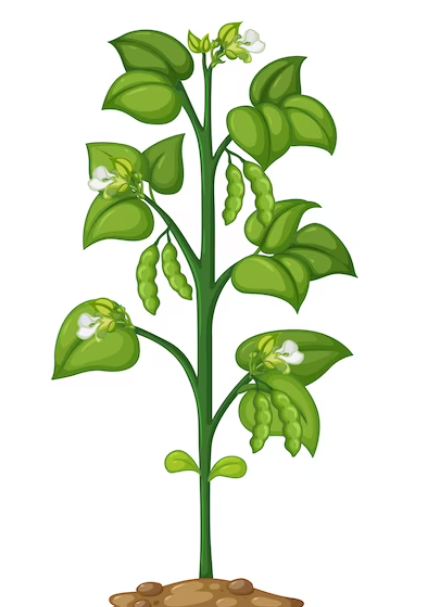  n = 5 | 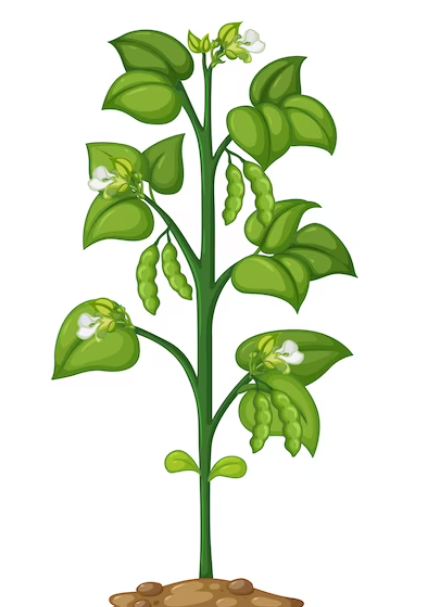  n = 5 |
|  | 0.5 mg/L | 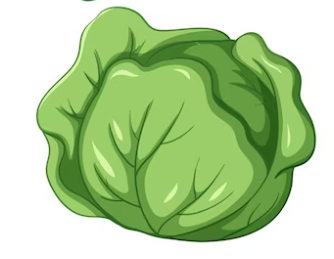  n = 5 | 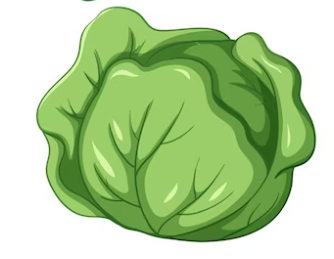  n = 5 | 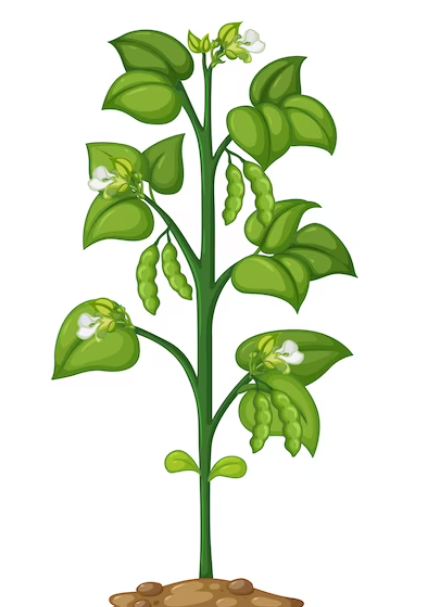  n = 5 | 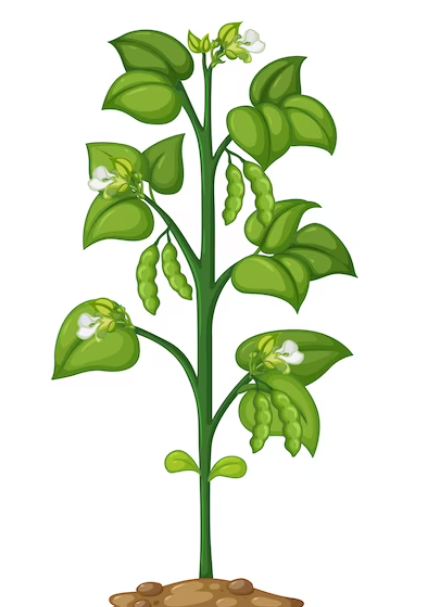  n = 5 | 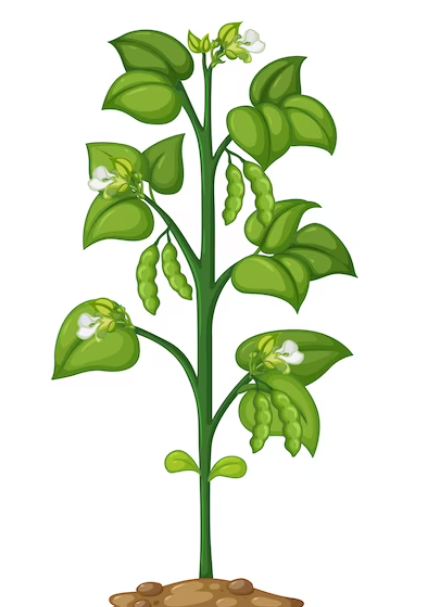  n = 5 |

**Table S2** Experimental design with the As treatments applied and the number of plants harvested at different growth stages in lettuce and bean.

| **Parameters** | **Values** | **Reference** |
| --- | --- | --- |
| Arsenic concentration in vegetable (C) | Varied depending on vegetable (mg/kg D.W.) | Present study |
| Arsenic reference dose (RfD) | 0.0003 mg/kg/day | USEPA 2012 |
| Arsenic cancer slope factor (CSF) | Adult: 1.5 kg day/mg  Child: 4.5 kg day/mg | USEPA 2012  Rehman et al. (2016) |
| Body weight (B.W.) | Adult: 70.8 kg  Child: 32.7 kg | Walpole et al. (2012)  Biswas et al. (2019) |
| Conversion factor (Cf) | 0.085 | Rehman et al. (2016) |
| Daily intake rate of vegetables (IR) | Adult: 500 g FW  Child: 300 g FW | Based on WHO recommendation |
| Exposure duration (ED) | Adult: 70 years  Child: 10 years | Rehman et al. (2016)  Sharma et al. (2016) |
| Exposure frequency (EF) | 365 days/year | Sharma et al. (2016) |
| Life Expectancy (LE) | Adult: 25,550 days  Child: 3650 days | Rehman et al. (2016)  Sharma et al. (2016) |

**Table S3** Parameters used for the calculation of health risk assessment

| pH | 7.71 |
| --- | --- |
| Sand (%) | 81 |
| Silt (%) | 13 |
| Clay (%) | 6 |
| Organic matter (w/w%) | 0.50 |
| CaCO_3_ (w/w%) | 16.1 |
| CEC (Na meq/100g) | 4.8 |
| AL-K_2_O (mg/kg) | 48 |
| AL-P_2_O_5_ (mg/kg) | 129 |
| Total nitrogen (w/w %) | 0.067 |
| NH_4_-N (mg/kg) | 3.2 |
| NO_3_-N (mg/kg) | 3.2 |
| Pseudo-total As (mg/kg) | 3.5 |
| Water-soluble As (mg/kg) | 0.023 |
| \| Al (mg/kg) \|  \| \| --- \| --- \| | 0.79 |
| \| Cu (mg/kg) \|  \| \| --- \| --- \| | 4.43 |
| \| Zn (mg/kg) \| \| --- \| | 8.17 |

**Table S4** Physical and chemical parameters of the soil

**References**

Biswas A, Swain S, Chowdhury NR, et al (2019) Arsenic contamination in Kolkata metropolitan city: perspective of transportation of agricultural products from arsenic-endemic areas. Environ Sci Pollut Res 26:22929–22944. https://doi.org/10.1007/s11356-019-05595-z

Rehman ZU, Khan S, Qin K, et al (2016) Quantification of inorganic arsenic exposure and cancer risk via consumption of vegetables in southern selected districts of Pakistan. Sci Total Environ 550:321–329. https://doi.org/10.1016/j.scitotenv.2016.01.094

Sandil S, Dobosy P, Kröpfl K, et al (2019) Effect of irrigation water containing arsenic on elemental composition of bean and lettuce plants cultivated in calcareous sandy soil. Food Prod Process Nutr 1:14. https://doi.org/10.1186/s43014-019-0014-3

Sharma S, Kaur J, Nagpal AK, Kaur I (2016) Quantitative assessment of possible human health risk associated with consumption of arsenic contaminated groundwater and wheat grains from Ropar Wetand and its environs. Environ Monit Assess 188:. https://doi.org/10.1007/s10661-016-5507-9

USEPA. 2012. Integrated risk information system (IRIS). Available at: https://www.epa.gov/iris

Walpole SC, Prieto-Merino D, Edwards P, et al (2012) The weight of nations: An estimation of adult human biomass. BMC Public Health 12:. https://doi.org/10.1186/1471-2458-12-439
